# 3-Benzyl-6-bromo-1*H*-imidazo[4,5-*b*]pyridin-2(3*H*)-one

**DOI:** 10.1107/S1600536813013780

**Published:** 2013-05-25

**Authors:** Youssef Kandri Rodi, Amal Haoudi, Frédéric Capet, Ahmed Mazzah, El Mokhtar Essassi, Lahcen El Ammari

**Affiliations:** a Laboratoire de Chimie Organique Appliquée, Université Sidi Mohamed Ben Abdallah, Faculté des Sciences et Techniques, Route d’Immouzzer, BP 2202 Fès, Morocco; b Unité de Catalyse et de Chimie du Solide (UCCS), UMR 8181, Ecole Nationale Supérieure de Chimie de Lille, France; c USR 3290 Miniaturisation pour l’Analyse, la Synthèse et la Protéomique, 59655 Villeneuve d’Ascq Cedex, Université Lille 1, France; d Laboratoire de Chimie Organique Hétérocyclique, URAC 21, Pôle de Compétences Pharmacochimie, Université Mohammed V-Agdal, BP 1014 Avenue, Ibn Batouta, Rabat , Morocco; e Laboratoire de Chimie du Solide Appliquée, Faculté des Sciences, Université Mohammed V-Agdal, Avenue Ibn Battouta, BP 1014, Rabat, Morocco

## Abstract

The fused imidazole and pyridine rings in the title compound, C_13_H_10_BrN_3_O, are linked to a benzyl group. The fused ring system is essentially planar, the largest deviation from the mean plane being 0.006 (2) Å. The phenyl ring is not coplanar with the fused ring system, as indicated by the dihedral angle of 67.04 (12)°. In the crystal, mol­ecules are linked by pairs of N—H⋯O hydrogen bonds, forming inversion dimers.

## Related literature

For the biological activity of imidazo­pyridine derivatives, see: Chen & Dost (1992 ▶); Cappelli *et al.* (2006 ▶); Weier *et al.* (1994 ▶); Kulkarni & Newman (2007 ▶); Bavetsias *et al.* (2007 ▶, 2010 ▶).
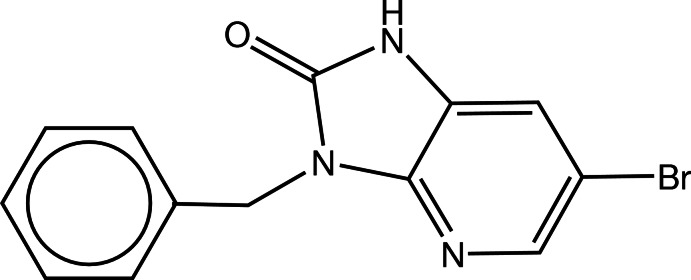


## Experimental

### 

#### Crystal data

C_13_H_10_BrN_3_O
*M* *_r_* = 304.15
Triclinic, 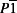

*a* = 4.2399 (2) Å
*b* = 10.4463 (4) Å
*c* = 14.5144 (6) Å
α = 107.611 (2)°
β = 90.628 (3)°
γ = 99.784 (3)°
*V* = 602.49 (4) Å^3^
*Z* = 2
Mo *K*α radiation
μ = 3.40 mm^−1^
*T* = 296 K
0.26 × 0.19 × 0.02 mm

#### Data collection

Bruker APEXII CCD diffractometer
Absorption correction: multi-scan (*SADABS*; Bruker, 2009 ▶) *T*_min_ = 0.472, *T*_max_ = 0.935
13819 measured reflections
2772 independent reflections
2169 reflections with *I* > 2σ(*I*)
*R*_int_ = 0.026

#### Refinement

*R*[*F* ^2^ > 2σ(*F* ^2^)] = 0.033
*wR*(*F* ^2^) = 0.085
*S* = 1.06
2772 reflections
163 parameters
H-atom parameters constrained
Δρ_max_ = 0.56 e Å^−3^
Δρ_min_ = −0.55 e Å^−3^

### 

Data collection: *APEX2* (Bruker, 2009 ▶); cell refinement: *SAINT-Plus* (Bruker, 2009 ▶); data reduction: *SAINT-Plus*; program(s) used to solve structure: *SHELXS97* (Sheldrick, 2008 ▶); program(s) used to refine structure: *SHELXL97* (Sheldrick, 2008 ▶); molecular graphics: *ORTEP-3 for Windows* (Farrugia, 2012 ▶); software used to prepare material for publication: *PLATON* (Spek, 2009 ▶) and *publCIF* (Westrip, 2010 ▶).

## Supplementary Material

Crystal structure: contains datablock(s) I, global. DOI: 10.1107/S1600536813013780/fj2630sup1.cif

Structure factors: contains datablock(s) I. DOI: 10.1107/S1600536813013780/fj2630Isup2.hkl

Supplementary material file. DOI: 10.1107/S1600536813013780/fj2630Isup3.cml

Additional supplementary materials:  crystallographic information; 3D view; checkCIF report

## Figures and Tables

**Table 1:** Hydrogen-bond geometry (Å, °)

*D*—H⋯*A*	*D*—H	H⋯*A*	*D*⋯*A*	*D*—H⋯*A*
N3—H14⋯O1^i^	0.86	1.95	2.789 (3)	166

Symmetry code: (i) 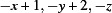.

**Fig. 1.**
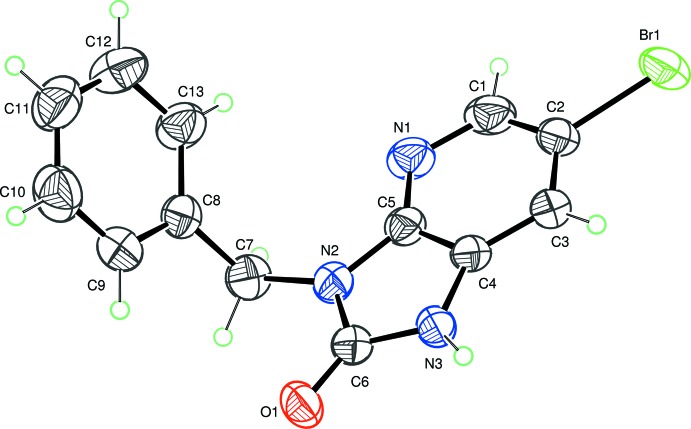
: Molecular plot the title compound with the atom-labelling scheme. Displacement ellipsoids are drawn at the 50% probability level. H atoms are represented as small circles.

**Fig. 2.**
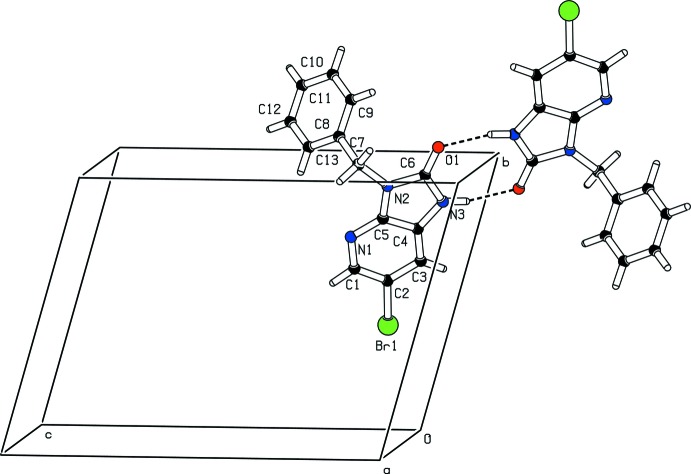
: Intermolecular interactions in the title compound building a dimers. Hydrogen bonds are shown as dashed lines.

## supplementary crystallographic information

### Comment

The imidazopyridine moieties are important pharmacophores, which have proven to be useful for a number of biologically relevant targets. The compounds derived from the imidazopyridine system have recently been evaluated as antagonists of various biological receptors, including angiotensin-II (Chen *et al.*, 1992; Cappelli *et al.*, 2006), platelet activating factor (Weier *et al.*, 1994), and metabotropic glutamate subtype V (Kulkarni *et al.*, 2007). Recently, a series of imidazo[4,5-*b*] pyridine derivatives as orally bioavailable Aurora A inhibitors with excellent potencies were reported (Bavetsias *et al.*, 2007; Bavetsias *et al.*, 2010) Hence, the synthesis of imidazo[4,5-*b*]pyridine derivatives is currently of great interest. Despite the importance of these intermediates, the methodology available for the synthesis was generally target-specific and restrictive in their scope.

Here, we wish to report a novel route leading to 3-benzyl-6-bromo-1,3- dihydro-imidazo[4,5-*b*]pyridin-2-one. We have checked the action of benzyl chloride towards 6-bromo-1,3-dihydro-imidazo[4,5 - b-]pyridin-2- one using K_2_CO_3_ as base (schem1).

The molecule of title compound, 3-benzyl-6-bromo-1,3-dihydro-imidazo [4,5-*b*]pyridin-2-one, build up from two fused five- and six-membered rings liked to a benzyl cycle as shown in Fig. 1. The fused rings system (N1N2N3 C1 to C6) is essentially planar with the largest deviation from the mean plane being -0.006 (2) A° at C5 atom. The dihedral angle between the benzyl cycle and the fused imidazole and pyridine rings is of 67.04 (12) °. In the crystal, the molecules are linked by N3–H14···O1 hydrogen bond in the way to build dimers as shown in Fig. 2 and Table 2.

### Experimental

To a stirred solution of 6-bromo-1,3-dihydro-imidazo[4,5 - b-]pyridin-2-one (0.2 g; 93.4 mmol), K_2_CO_3_ (0.38 g; 2.8 mmol), and tetra n-butylammonium bromide (0.03 g; 9.34 10 ^-5^ mol) in DMF, benzyl chloride (95 mmol) was added dropwise. Later the mixture was heated under reflux for 24 h. After completion of reaction (monitored by TLC), the salt was filtered and the solvent was removed under reduced pressure. The resulting residue was purified by column chromatography on silica gel using (ethyl acetate/hexane) (1/2) as eluent. The yield of the reaction is of 85%. Crystals were isolated after the solvent (hexane / acetate d'ethyle: 1/1) was allowed to evaporate.

### Refinement

All H atoms could be located in a difference Fourier map. However, they were placed in calculated positions with C—H = 0.93 Å (aromatic), N—H = 0.86 and C—H = 0.97 Å (methylene) and refined as riding on their parent atoms with *U*_iso_(H) = 1.2 *U*_eq_ (C, N).

### Crystal data

**Table:**

C_13_H_10_BrN_3_O	*Z* = 2
*M**_r_* = 304.15	*F*(000) = 304
Triclinic, *P*1	*D*_x_ = 1.677 Mg m^−^^3^
Hall symbol: -P 1	Melting point: 358 K
*a* = 4.2399 (2) Å	Mo *K*α radiation, λ = 0.71073 Å
*b* = 10.4463 (4) Å	Cell parameters from 2772 reflections
*c* = 14.5144 (6) Å	θ = 1.5–27.5°
α = 107.611 (2)°	µ = 3.40 mm^−^^1^
β = 90.628 (3)°	*T* = 296 K
γ = 99.784 (3)°	Platelet, colourless
*V* = 602.49 (4) Å^3^	0.26 × 0.19 × 0.02 mm

### Data collection

**Table:**

Bruker APEXII CCD diffractometer	2772 independent reflections
Radiation source: microfocus source	2169 reflections with *I* > 2σ(*I*)
Graphite monochromator	*R*_int_ = 0.026
φ and ω scans	θ_max_ = 27.5°, θ_min_ = 1.5°
Absorption correction: multi-scan (*SADABS*; Bruker, 2009)	*h* = −5→5
*T*_min_ = 0.472, *T*_max_ = 0.935	*k* = −13→13
13819 measured reflections	*l* = −18→18

### Refinement

**Table:**

Refinement on *F*^2^	Primary atom site location: structure-invariant direct methods
Least-squares matrix: full	Secondary atom site location: difference Fourier map
*R*[*F*^2^ > 2σ(*F*^2^)] = 0.033	Hydrogen site location: difference Fourier map
*wR*(*F*^2^) = 0.085	H-atom parameters constrained
*S* = 1.06	*w* = 1/[σ^2^(*F*_o_^2^) + (0.0374*P*)^2^ + 0.3201*P*] where *P* = (*F*_o_^2^ + 2*F*_c_^2^)/3
2772 reflections	(Δ/σ)_max_ < 0.001
163 parameters	Δρ_max_ = 0.56 e Å^−^^3^
0 restraints	Δρ_min_ = −0.55 e Å^−^^3^

### Special details

**Table:**

Geometry. All e.s.d.'s (except the e.s.d. in the dihedral angle between two l.s. planes) are estimated using the full covariance matrix. The cell e.s.d.'s are taken into account individually in the estimation of e.s.d.'s in distances, angles and torsion angles; correlations between e.s.d.'s in cell parameters are only used when they are defined by crystal symmetry. An approximate (isotropic) treatment of cell e.s.d.'s is used for estimating e.s.d.'s involving l.s. planes.
Refinement. Refinement of *F*^2^ against all reflections. The weighted *R*-factor *wR* and goodness of fit *S* are based on *F*^2^, conventional *R*-factors *R* are based on *F*, with *F* set to zero for negative *F*^2^. The threshold expression of *F*^2^ > σ(*F*^2^) is used only for calculating *R*-factors(gt) *etc*. and is not relevant to the choice of reflections for refinement. *R*-factors based on *F*^2^ are statistically about twice as large as those based on *F*, and *R*- factors based on all data will be even larger.

### Fractional atomic coordinates and isotropic or equivalent isotropic displacement parameters (Å^2^)

**Table:**

	*x*	*y*	*z*	*U*_iso_*/*U*_eq_	
Br1	0.26054 (8)	0.40434 (3)	0.14153 (2)	0.06526 (14)	
C1	0.6829 (7)	0.6528 (3)	0.2362 (2)	0.0522 (7)	
H1	0.7269	0.6048	0.2779	0.063*	
C2	0.4658 (7)	0.5871 (3)	0.15899 (19)	0.0463 (6)	
C3	0.3894 (6)	0.6511 (3)	0.09261 (18)	0.0448 (6)	
H3	0.2437	0.6073	0.0395	0.054*	
C4	0.5450 (6)	0.7837 (2)	0.11128 (16)	0.0373 (5)	
C5	0.7610 (6)	0.8423 (2)	0.19198 (16)	0.0379 (5)	
C6	0.7491 (6)	0.9986 (2)	0.11485 (16)	0.0387 (5)	
C7	1.1131 (6)	1.0750 (3)	0.26618 (18)	0.0433 (6)	
H7A	1.2362	1.1371	0.2363	0.052*	
H7B	1.2615	1.0289	0.2896	0.052*	
C8	0.9545 (5)	1.1565 (2)	0.35104 (16)	0.0381 (5)	
C9	0.8773 (7)	1.2797 (3)	0.3530 (2)	0.0503 (6)	
H9	0.9188	1.3126	0.3008	0.060*	
C10	0.7386 (8)	1.3551 (3)	0.4320 (2)	0.0644 (8)	
H10	0.6870	1.4385	0.4327	0.077*	
C11	0.6766 (8)	1.3071 (4)	0.5096 (2)	0.0647 (8)	
H11	0.5865	1.3586	0.5632	0.078*	
C12	0.7474 (8)	1.1844 (4)	0.5076 (2)	0.0656 (8)	
H12	0.7029	1.1513	0.5596	0.079*	
C13	0.8849 (7)	1.1088 (3)	0.42872 (19)	0.0536 (7)	
H13	0.9313	1.0245	0.4278	0.064*	
N1	0.8370 (6)	0.7832 (2)	0.25554 (15)	0.0479 (5)	
N2	0.8850 (5)	0.9739 (2)	0.19325 (14)	0.0385 (4)	
N3	0.5415 (5)	0.8819 (2)	0.06533 (14)	0.0412 (5)	
H14	0.4262	0.8712	0.0135	0.049*	
O1	0.8058 (5)	1.10622 (18)	0.09525 (13)	0.0507 (4)	

### Atomic displacement parameters (Å^2^)

**Table:**

	*U*^11^	*U*^22^	*U*^33^	*U*^12^	*U*^13^	*U*^23^
Br1	0.0925 (3)	0.04191 (17)	0.0673 (2)	0.00734 (15)	0.02819 (17)	0.02745 (14)
C1	0.0752 (19)	0.0483 (15)	0.0458 (15)	0.0241 (14)	0.0182 (14)	0.0259 (13)
C2	0.0615 (16)	0.0352 (13)	0.0453 (14)	0.0094 (12)	0.0203 (12)	0.0159 (11)
C3	0.0572 (15)	0.0369 (13)	0.0378 (13)	0.0036 (11)	0.0109 (11)	0.0103 (11)
C4	0.0468 (13)	0.0346 (12)	0.0323 (11)	0.0084 (10)	0.0083 (10)	0.0121 (10)
C5	0.0437 (13)	0.0369 (12)	0.0352 (12)	0.0119 (10)	0.0100 (10)	0.0113 (10)
C6	0.0460 (13)	0.0358 (12)	0.0345 (12)	0.0060 (10)	0.0040 (10)	0.0119 (10)
C7	0.0357 (12)	0.0496 (14)	0.0428 (13)	0.0047 (11)	−0.0023 (10)	0.0134 (12)
C8	0.0321 (12)	0.0419 (13)	0.0355 (12)	−0.0009 (10)	−0.0081 (9)	0.0094 (10)
C9	0.0581 (16)	0.0427 (14)	0.0511 (15)	0.0044 (12)	0.0053 (12)	0.0185 (12)
C10	0.074 (2)	0.0440 (16)	0.074 (2)	0.0150 (15)	0.0095 (17)	0.0142 (15)
C11	0.069 (2)	0.065 (2)	0.0518 (17)	0.0155 (16)	0.0109 (14)	0.0036 (15)
C12	0.080 (2)	0.079 (2)	0.0422 (15)	0.0184 (18)	0.0089 (14)	0.0238 (15)
C13	0.0682 (18)	0.0556 (17)	0.0426 (14)	0.0191 (14)	0.0021 (13)	0.0194 (13)
N1	0.0618 (13)	0.0493 (13)	0.0397 (11)	0.0181 (11)	0.0045 (10)	0.0199 (10)
N2	0.0435 (11)	0.0369 (10)	0.0337 (10)	0.0055 (9)	−0.0001 (8)	0.0099 (8)
N3	0.0544 (12)	0.0349 (10)	0.0324 (10)	−0.0006 (9)	−0.0056 (9)	0.0125 (8)
O1	0.0670 (12)	0.0366 (9)	0.0466 (10)	−0.0043 (8)	−0.0091 (9)	0.0177 (8)

### Geometric parameters (Å, º)

**Table:**

Br1—C2	1.898 (3)	C7—C8	1.507 (3)
C1—N1	1.350 (4)	C7—H7A	0.9700
C1—C2	1.368 (4)	C7—H7B	0.9700
C1—H1	0.9300	C8—C9	1.372 (4)
C2—C3	1.392 (4)	C8—C13	1.380 (4)
C3—C4	1.373 (3)	C9—C10	1.381 (4)
C3—H3	0.9300	C9—H9	0.9300
C4—N3	1.385 (3)	C10—C11	1.375 (5)
C4—C5	1.392 (3)	C10—H10	0.9300
C5—N1	1.320 (3)	C11—C12	1.357 (5)
C5—N2	1.380 (3)	C11—H11	0.9300
C6—O1	1.227 (3)	C12—C13	1.378 (4)
C6—N3	1.367 (3)	C12—H12	0.9300
C6—N2	1.381 (3)	C13—H13	0.9300
C7—N2	1.458 (3)	N3—H14	0.8600
			
N1—C1—C2	123.9 (2)	C9—C8—C7	120.9 (2)
N1—C1—H1	118.1	C13—C8—C7	120.5 (2)
C2—C1—H1	118.1	C8—C9—C10	120.6 (3)
C1—C2—C3	121.7 (2)	C8—C9—H9	119.7
C1—C2—Br1	119.55 (19)	C10—C9—H9	119.7
C3—C2—Br1	118.8 (2)	C11—C10—C9	120.0 (3)
C4—C3—C2	115.1 (2)	C11—C10—H10	120.0
C4—C3—H3	122.5	C9—C10—H10	120.0
C2—C3—H3	122.5	C12—C11—C10	119.8 (3)
C3—C4—N3	133.9 (2)	C12—C11—H11	120.1
C3—C4—C5	119.2 (2)	C10—C11—H11	120.1
N3—C4—C5	106.8 (2)	C11—C12—C13	120.2 (3)
N1—C5—N2	125.9 (2)	C11—C12—H12	119.9
N1—C5—C4	126.6 (2)	C13—C12—H12	119.9
N2—C5—C4	107.5 (2)	C12—C13—C8	120.7 (3)
O1—C6—N3	127.5 (2)	C12—C13—H13	119.6
O1—C6—N2	125.6 (2)	C8—C13—H13	119.6
N3—C6—N2	107.0 (2)	C5—N1—C1	113.6 (2)
N2—C7—C8	113.10 (19)	C5—N2—C6	109.01 (19)
N2—C7—H7A	109.0	C5—N2—C7	126.9 (2)
C8—C7—H7A	109.0	C6—N2—C7	124.1 (2)
N2—C7—H7B	109.0	C6—N3—C4	109.67 (19)
C8—C7—H7B	109.0	C6—N3—H14	125.2
H7A—C7—H7B	107.8	C4—N3—H14	125.2
C9—C8—C13	118.6 (2)		
			
N1—C1—C2—C3	0.8 (4)	C7—C8—C13—C12	178.3 (3)
N1—C1—C2—Br1	−178.9 (2)	N2—C5—N1—C1	−179.1 (2)
C1—C2—C3—C4	−0.6 (4)	C4—C5—N1—C1	0.3 (4)
Br1—C2—C3—C4	179.11 (17)	C2—C1—N1—C5	−0.6 (4)
C2—C3—C4—N3	179.7 (2)	N1—C5—N2—C6	179.6 (2)
C2—C3—C4—C5	0.3 (3)	C4—C5—N2—C6	0.2 (2)
C3—C4—C5—N1	−0.1 (4)	N1—C5—N2—C7	−2.6 (4)
N3—C4—C5—N1	−179.7 (2)	C4—C5—N2—C7	178.0 (2)
C3—C4—C5—N2	179.3 (2)	O1—C6—N2—C5	179.4 (2)
N3—C4—C5—N2	−0.3 (3)	N3—C6—N2—C5	−0.1 (3)
N2—C7—C8—C9	−93.6 (3)	O1—C6—N2—C7	1.5 (4)
N2—C7—C8—C13	86.6 (3)	N3—C6—N2—C7	−177.9 (2)
C13—C8—C9—C10	1.2 (4)	C8—C7—N2—C5	−86.5 (3)
C7—C8—C9—C10	−178.5 (3)	C8—C7—N2—C6	91.0 (3)
C8—C9—C10—C11	0.0 (5)	O1—C6—N3—C4	−179.6 (2)
C9—C10—C11—C12	−1.1 (5)	N2—C6—N3—C4	−0.1 (3)
C10—C11—C12—C13	0.9 (5)	C3—C4—N3—C6	−179.2 (3)
C11—C12—C13—C8	0.4 (5)	C5—C4—N3—C6	0.3 (3)
C9—C8—C13—C12	−1.4 (4)		

### Hydrogen-bond geometry (Å, º)

**Table:**

*D*—H···*A*	*D*—H	H···*A*	*D*···*A*	*D*—H···*A*
N3—H14···O1^i^	0.86	1.95	2.789 (3)	166

Symmetry code: (i) −*x*+1, −*y*+2, −*z*.
